# Probing Host Pathogen Cross-Talk by Transcriptional Profiling of Both *Mycobacterium tuberculosis* and Infected Human Dendritic Cells and Macrophages

**DOI:** 10.1371/journal.pone.0001403

**Published:** 2008-01-02

**Authors:** Ludovic Tailleux, Simon J. Waddell, Mattia Pelizzola, Alessandra Mortellaro, Michael Withers, Antoine Tanne, Paola Ricciardi Castagnoli, Brigitte Gicquel, Neil G. Stoker, Philip D. Butcher, Maria Foti, Olivier Neyrolles

**Affiliations:** 1 Institut Pasteur, Unit of Mycobacterial Genetics, Paris, France; 2 Medical Microbiology, Division of Cellular and Molecular Medicine, St. George's University of London, London, United Kingdom; 3 Department of Biotechnology and Bioscience, University of Milan-Bicocca, Milan, Italy; 4 Department of Pathology and Infectious Diseases, Royal Veterinary College, London, United Kingdom; New York University School of Medicine, United States of America

## Abstract

**Background:**

Transcriptional profiling using microarrays provides a unique opportunity to decipher host pathogen cross-talk on the global level. Here, for the first time, we have been able to investigate gene expression changes in both *Mycobacterium tuberculosis*, a major human pathogen, and its human host cells, macrophages and dendritic cells.

**Methodology/Principal Findings:**

In addition to common responses, we could identify eukaryotic and microbial transcriptional signatures that are specific to the cell type involved in the infection process. In particular *M. tuberculosis* shows a marked stress response when inside dendritic cells, which is in accordance with the low permissivity of these specialized phagocytes to the tubercle bacillus and to other pathogens. In contrast, the mycobacterial transcriptome inside macrophages reflects that of replicating bacteria. On the host cell side, differential responses to infection in macrophages and dendritic cells were identified in genes involved in oxidative stress, intracellular vesicle trafficking and phagosome acidification.

**Conclusions/Significance:**

This study provides the proof of principle that probing the host and the microbe transcriptomes simultaneously is a valuable means to accessing unique information on host pathogen interactions. Our results also underline the extraordinary plasticity of host cell and pathogen responses to infection, and provide a solid framework to further understand the complex mechanisms involved in immunity to *M. tuberculosis* and in mycobacterial adaptation to different intracellular environments.

## Introduction

Co-evolution of microbes and the immune system has resulted in the selection of sophisticated mechanisms, which may provide advantages to the host or to the microbe, and ultimately result in resistance or susceptibility to infectious disease. The use of both human and pathogen microarrays in time-course experiments may allow the activities of host and pathogen to be measured simultaneously, and might show how gene expression changes in the host correlate with those observed in the microorganism and *vice versa*. A detailed comprehension of the common responses is likely to give insight into the basic mechanisms governing host-pathogen cross-talk, whereas genes that are modulated in a cell-specific manner may provide information about specific gene expression programs initiated upon pathogen encounter. These studies will ultimately allow the dissection of regulatory networks, which underlie the transcriptional response to infection [1], [2], [3], [4].

Here we sought to use microarray technology to decipher simultaneously transcriptional changes in a human pathogen of primary public health importance, *Mycobacterium tuberculosis*, and in its main host cells, macrophages (Mφs) and dendritic cells (DCs) throughout infection.

A major virulence feature of the tuberculosis (TB) bacillus relies on the mechanisms it has evolved to parasitize host phagocytes [5], [6]. DCs and Mφs are continuously produced from common hematopoietic stem cells within the bone marrow ; both cell types are central to anti-mycobacterial immunity and to TB pathogenesis, yet they serve distinct roles during the infection process. While alveolar Mφs act as sentinel cells by engulfing foreign inhaled particles by active phagocytosis and play a scavenger function, they are poor activators of naive T cells. In contrast, DCs are able to initiate and modulate adaptive immune responses through recognition and phagocytosis of pathogens at the sites of infection, and through subsequent cytokine secretion and migration to the draining lymph nodes where they process and present antigens to naive lymphocytes. The outcome of host cell and mycobacterial interactions most likely depends on differential molecular events, a snapshot of which may be measured in the changing transcriptional profiles of Mφs and DCs, which we have investigated here. Previously, a number of important studies have been published dealing with global gene expression profiling of *M. tuberculosis*-infected mouse [7] or human [8], [9], [10], [11] Mφs and DCs [8], and with mycobacterial transcriptome analysis in mouse [12] or human Mφs [13], or in mouse [14], [15] or human [16] lung tissue samples. Here were able to compare the transcriptional responses upon mycobacterial infection both in the pathogen and in infected Mφs and DCs derived from the same donors. Using microarrays designed to probe the human or the mycobacterial transcriptomes, we could follow these changes simultaneously over time-course experiments. Our results identify a core set of genes that respond similarly in Mφs and DCs upon *M. tuberculosis* infection, as well as cell-type specific gene expression patterns; on the microbial side, mycobacteria exhibit both a common response to Mφ and DC infection, as well as differential responses to the two cell types. In particular, we could identify a clear mycobacterial stress response signature in DCs, which is in line with previous findings on the low replication rate of bacilli inside these cells [17]. In contrast the mycobacterial transcriptome in Mφs reflects that of intracellularly replicating bacteria.

Altogether, these results highlight that global and simultaneous gene expression profiling of both the host and the pathogen is a useful means of accessing information on host pathogen interactions; our study also provides a solid framework for further understanding host pathogen interactions in TB.

## Results

### *M. tuberculosis* induces differential responses in human Mφs and DCs

**Figure 1:**
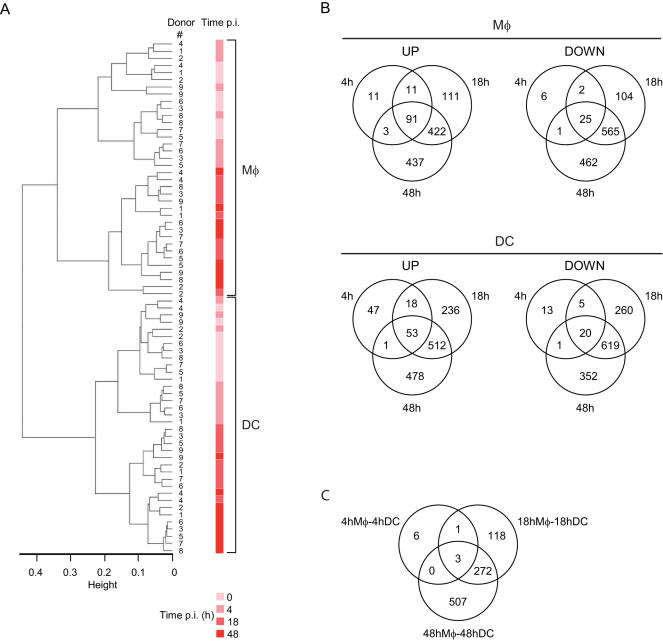
Transcriptional differences between *M. tuberculosis*-infected Mφs and DCs. (A) Hierarchical clustering of arrays indicating the donor # (1–9), the time of infection (0, 4, 18, 48 h) and the cell type (Mφs vs DCs). (B) Venn diagrams illustrating the number of up- and down-regulated genes in Mφs (upper panels) and DCs (lower panels) after 4, 18, or 48 h infection, as compared to basal expression levels at the time of infection. (C) Venn diagram showing the number of genes differently modulated in the direct comparison of Mφs and DCs after 4, 18, and 48 h infection.

Human monocyte-derived Mφs and DCs were infected by *M. tuberculosis* for 4, 18 or 48 hours, and cellular transcriptomes were analyzed as compared to the reference transcriptome at the time of infection (time-point 0). Comprehensive gene expression profiles of 9 independent healthy donors were generated with high-density oligonucleotide human arrays with 22,283 probe sets, which in total interrogated the expression levels of approximately 18,400 transcripts and variants, including 14,500 well-characterized human genes. Using unsupervised hierarchical cluster analysis with 11,262 probe sets we identified the differences in gene expression between DCs and Mφs, which readily distinguished the two groups. As shown in Figure 1A, Mφs and DCs were found to group into two classes independently of the time-point and of the donor analyzed, thus identifying distinct responses to infection. Moreover, within the same cluster early (0–4 hours) and late (18–48 hours) molecular signatures could be identified. Altogether, these results clearly show that host cell responses depend mainly on the cell type and the duration of infection, and that donor-to-donor variability only weakly influences the response profiles.

A subsequent statistical analysis was applied to select the genes associated with infection. Using the Limma Bioconductor library, 2,251 and 2,615 probe sets were found to be significantly differentially regulated in Mφs and DCs respectively (Figure 1B). The sets of genes modulated upon infection in Mφs and DCs mostly overlapped, yet Mφs and DCs showed clear differences in gene regulation during infection, especially at time-points 18 h and 48 h (Figure 1C). As a validation of our data, we looked at selected examples of genes described as showing altered expression in previous studies. Thus *CD1a-c*, *CD83*, and *interleukin* (*IL*)*-12p40* were modulated in DCs only (Suppl. Figure S1), as previously shown [8], [9], [18], [19], [20]. Conversely, expression of *IL-1β* and *IL-6* was induced mostly in Mφs, as previously reported for both mRNA and protein levels (Suppl. Figure S1 ; [18]).

### Functional annotation and clustering reveal a core response and cell type-specific signatures in *M. tuberculosis*-infected Mφs and DCs

**Figure 2:**
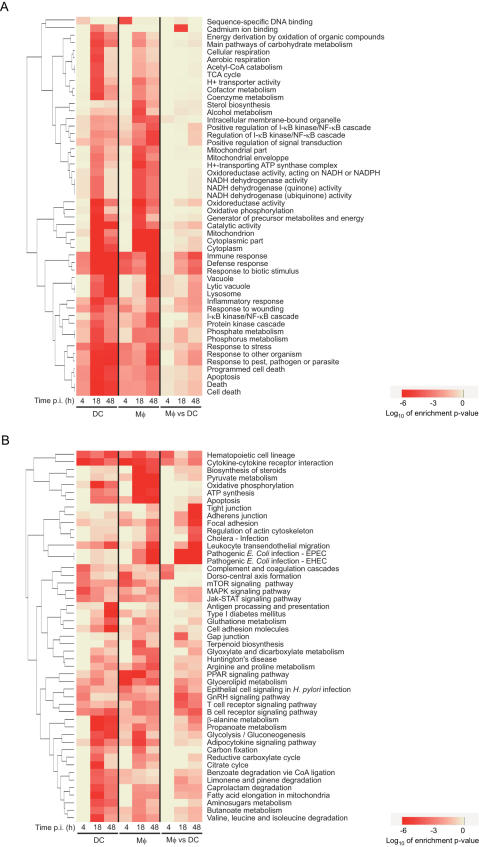
Clustering of functional categories altered in Mφs and DCs upon *M. tuberculosis* infection. The 50 top ranking GO (A) and KEGG (B) functional categories according to enrichment p-values of differentially expressed genes in Mφs and DCs at 4, 18 and 48 h post-infection as compared to baseline levels at the time of infection, and in DCs as compared to Mφs at 4, 18 and 48 h post-infection. The order of gene families was determined by hierarchical clustering. Annotation is given according to GO and KEGG nomenclatures. See the online GO and KEGG databases for further details.

In order to gain insight into the common and differential responses of Mφs and DCs to *M. tuberculosis* infection, we performed gene functional classification on the basis of the annotation resources provided by GeneOntology (GO) [21] and Kyoto Encyclopedia of Genes and Genomes (KEGG) [22], [23]. The annotation terms from each time point analysed were further clustered according to enrichment p-values (log_10_ of p-value ) in a functional summary as shown in Figure 2.

GO (Figure 2A) and KEGG (Figure 2B) annotations allowed us to identify gene families, whose expression is altered either in Mφs or in DCs or in both cell types upon infection. Annotation is given according to GO and KEGG nomenclatures. For instance, differentially expressed genes (DEGs) contained in the KEGG « Pathogenic *E. coli* infection-EPEC » and « Pathogenic *E. coli* infection-EHEC » categories include genes involved in sensing pathogens, such as *TLR4*, genes involved in intracellular signalling and trafficking, such as *CDC42EP3*, and other genes (see the online GO and KEGG databases for further details). The common gene clusters are mostly related to basic cellular processes such as carbohydrate and pyruvate metabolism, aerobic respiration and energy production.

**Figure 3:**
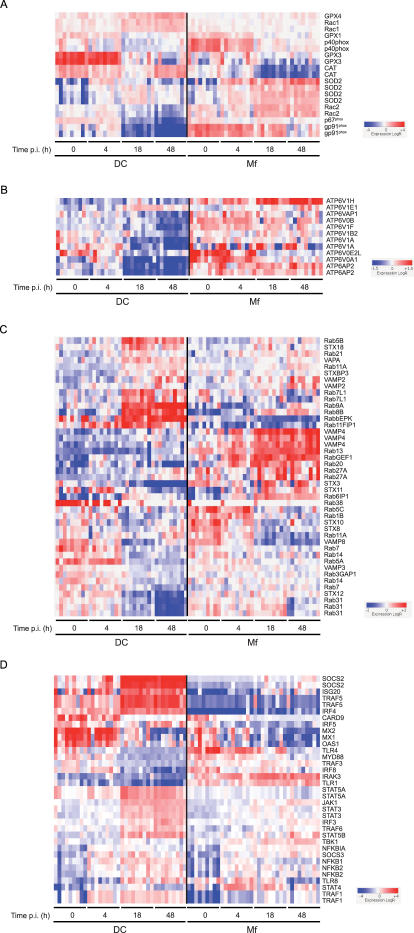
Differential regulation of genes involved in oxidative stress, vacuole acidification and intracellular trafficking in *M. tuberculosis*-infected Mφs and DCs. Red-blue display showing hierarchical clustering according to normalized expression levels of genes involved in (A) phagocyte oxidase assembly and resistance to oxidative stress, (B) v-ATPase production and phagosome acidification, (C) intracellular trafficking machinery and (D) IFN response and TLR-related pathways. Log_2_ ratios of absolute expression values divided by the median of each gene across all donors and conditions are reported according to the colour codes indicated.

**Figure 4:**
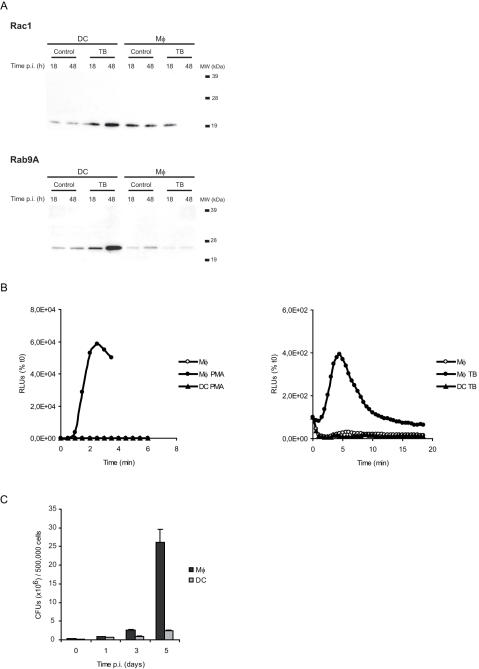
Validation of candidate genes and phenotypic characterization of *M. tuberculosis*-infected Mφs and DCs. (A) Western blotting validation of selected candidate genes *Rac1* and *Rab9A*. Each line contains 5 µg of total proteins. (B) Differential superoxide production expressed in relative light units (RLUs) by Mφs and DCs either treated with PMA (left panel), or infected with *M. tuberculosis* (right panel). (C) Differential multiplication of *M. tuberculosis* within human monocyte-derived Mφs and DCs.

Importantly, cell type-specific signatures are also identified. They are mainly annotated as being involved in the response to infection, as well as in cell motility and cytoskeleton remodelling (Figure 2). In particular, hierarchical clustering of expression patterns of genes related to oxidative stress (Figure 3A), and intracellular vesicle acidification (Figure 3B) and trafficking (Figure 3C) revealed profound differences in Mφ and DC responses to infection. As a selected example, the expression patterns of the small GTP-binding protein Rac isoforms 1 and 2 were the opposite in the two cell types : while Rac1 was induced in DCs and barely expressed in Mφs, which was confirmed by Western blotting (Figure 4A), the Rac2 isoform was induced in Mφs and not detected in DCs. Rac is part of the NADPH-dependent phagocyte oxidase (Phox), whose activity is prominently dependent on Rac2 rather than Rac1, at least in neutrophils [24], [25]. Other genes encoding Phox subunits, namely *p40^phox^*, *p67^phox^* and *gp91^phox^* were found to be preferentially expressed and/or induced in Mφs, as compared to in DCs, following infection (Figure 3A). Altogether, these results are in agreement with the general idea that Mφs are more prone to reactive oxygen species (ROS) production than DCs [26], which might limit phagosome acidification and promote antigenic peptide presentation [27]. In line with this view, we demonstrated that Mφs produce more superoxide anions, as compared to DCs, when treated with PMA or infected with *M. tuberculosis*, on the whole cell level (Figure 4B). In addition to its role in Phox activation, Rac acts as a molecular switch for signal transduction to regulate several cellular functions. The differential expression patterns of Rac1 and Rac2 in *M. tuberculosis*-infected Mφs and DCs might also have important consequences on intracellular trafficking of the bacillus and on various signalling cascades.

In contrast to the response to ROS, inducible nitric oxide synthase (*NOS2*) was not induced either in Mφs or in DCs, which is in accordance with previous reports in human Mφs [7], [11] and NO production could not be detected in either cell type (data not shown). Although this does not preclude for a role of NO in TB in humans, as attested by *in vitro* and *ex vivo* experiments [28], [29], this result is a clear discrepancy with that observed in mouse phagocytes, especially in Mφs, in which mycobacterial infection induces *NOS2* transcription and NO production [7]. Nevertheless, the roles of NO and reactive nitrogen intermediates in TB still remain to be fully elucidated [30].

Another example of differentially regulated genes, of interest in the context of *M. tuberculosis* infection, is the family of genes encoding the vesicular (v)-ATPase subunits (Figure 3B), as the mycobacterial phagosome has been reported to avoid fusion with v-ATPase-expressing intracellular vesicles in Mφs [31], [32]. The v-ATPase is composed of two main complexes, the V0 complex responsible for H^+^ import from the cytosol into the vesicular lumen, and the V1 complex responsible for ATP hydrolysis. The V0 and V1 complexes are formed of 6 and 8 subunits, respectively. The *ATP6V1H* gene, encoding the V1 50/57 kDa subunit, is strongly induced in Mφs upon infection, whereas it is barely expressed in DCs. Overall, genes in this class were differentially regulated in Mφs, but almost all were either not expressed or down-modulated in DCs. Our results suggest that *M. tuberculosis* infection induces a marked reprogramming of the v-ATPase-encoding genes in the Mφ, and pinpoints a profound difference in host cell endocytic machinery response to infection between Mφs and DCs. In line with this finding, we observed dramatic differences in modulation of genes encoding Rab GTPases and other modulators of intracellular trafficking in infected Mφs and DCs (Figure 3C). For instance *Rab9A* was found induced in DCs while it was barely expressed in Mφs (Figures 3C and 4A). Rab9 regulates vesicular trafficking from the trans-Golgi network (TGN) to the lysosomes through late endosomes [33] ; in particular this GTPase is required for transport of lysosomal hydrolases from the TGN to the lysosomes. The strong induction of Rab9 in DCs only likely reflects the specialized function of these cells in antigen processing and presentation and might interfere with phagosome biogenesis, which should be further investigated.

Apart from genes involved in intracellular trafficking and vesicle maturation, relevant differences between Mφ and DC responses to infection were detected in genes involved in intracellular signalling, in particular in interferon (IFN) response, Toll-like receptor (TLR) signalling and related signalling pathways [34] (Figure 3D). In general, DCs were more responsive than Mφs to infection, with more genes induced, such as *SOCS2*, *ISG20*, *TRAF5* and *IRF4* (Figure 3D). The very strong induction of *SOCS2* (suppressor of cytokine signalling 2) [35], [36] in DCs only, for instance, might have important consequences on the maturation and cytokine secretion profile of *M. tuberculosis*-infected DCs, which should be further explored.

Together, these results reveal that human DCs and Mφs respond differently to *M. tuberculosis* infection and allowed us to identify gene expression signatures specific to each cell type, which opens the way for further functional studies in host cell response to intracellular infection and to the immune response to TB.

### There are core and cell type-specific responses and cell type-specific *M. tuberculosis*

**Figure 5:**
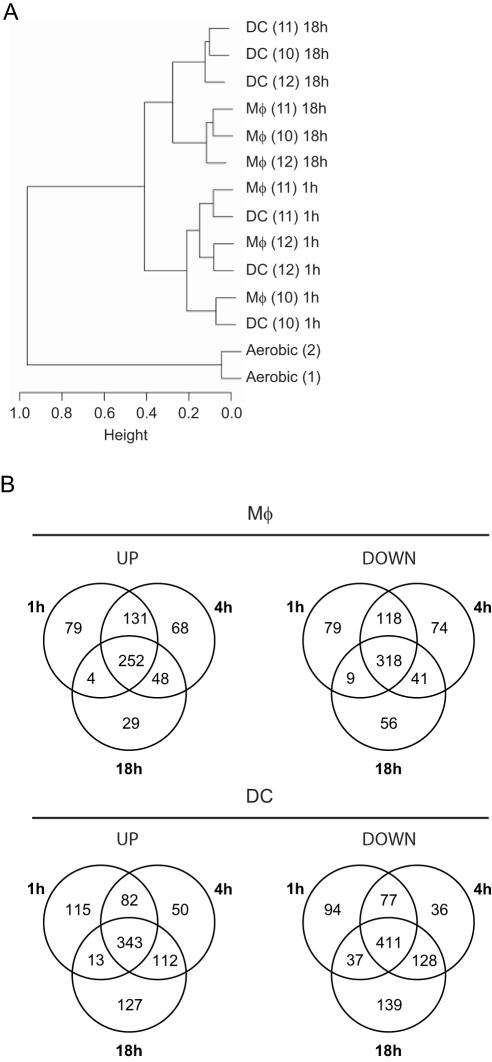
Differential mycobacterial response to Mφ and DC infection. (A) Hierarchical clustering of arrays indicating the donor # (10–12), the time of infection (1, 18 h) and the cell type (Mφs vs DCs). Aerobic indicates log-phase cultivated bacteria in axenic conditions. (B) Venn diagrams illustrating the number of up- and down-regulated mycobacterial genes in Mφs (upper panels) and DCs (lower panels) after 1, 4 and 18 h infection relative to aerobically cultivated bacilli.

In an attempt to further decipher host cell-mycobacteria interactions, we analyzed the changes in the mycobacterial transcriptome during infection of human Mφs and DCs. Phagocytes were derived and differentiated from monocytes of three independent healthy donors, and infected at a multiplicity of infection of 2–5 bacterium per cell for 1, 4 or 18 h. Mycobacterial RNA was extracted from infected cells using a differential lysis method previously described [12], [37], and amplified using a modified Eberwine T7-based system. Hierarchical clustering of the arrays (Figure 5A) clearly showed a mycobacterial response specific to an intracellular context as compared to *in vitro* log phase growth. Mycobacterial responses to DC or Mφ infection were also clearly distinguishable at the 18 h time point. This pattern reflects the changing cell-specific gene expression pattern of *M. tuberculosis* over time. Significantly differentially expressed genes were identified by comparing the intracellular mRNA profiles of *M. tuberculosis* with those derived from aerobically growing bacilli. The transcriptional patterns described below were also observed from infected Mφs and DCs extracted from two additional healthy donors as part of a pilot project. Transcriptome modification was more pronounced in *M. tuberculosis* extracted from DCs than in Mφs (with 1,764 *vs* 1,306 genes respectively differentially regulated relative to aerobic growth; Figure 5B). A common mycobacterial response to the two phagocytes was identified as well as cell-type specific signatures.

**Figure 6:**
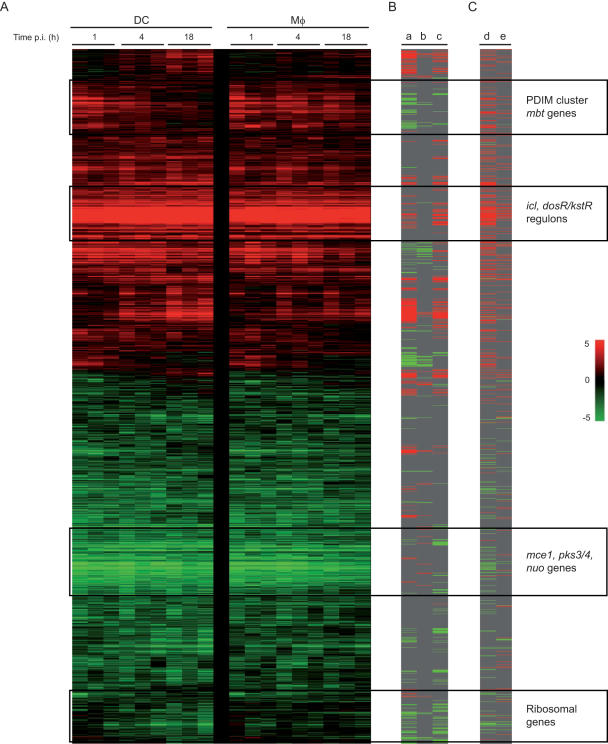
Functional and hierarchical clustering of the *M. tuberculosis* response to DCs and Mφs infection. (A) Red-green display showing 1,875 *M. tuberculosis* genes identified to be significantly differentially expressed at 1, 4 or 18 h in Mφs and DCs relative to aerobic *in vitro* growth. Genes are ordered in rows, conditions as columns. Red colouring indicates genes induced in intracellular *vs.* aerobic growth conditions (fold change); green colouring denotes repression. (B) Genes are highlighted that were significantly differentially regulated over time (18 h *vs*. 1 h) in the *M. tuberculosis* response to DCs (a) or Mφs (b), red colouring identifies genes induced with time, green repressed; together with (c) those genes identified to be over-expressed (red) or under-expressed (green) after infection of DCs compared to Mφs (DC18h *vs*. Mφ18h). (C) Genes previously identified in other intracellular studies as being modulated in specific conditions are marked, namely genes induced (red colouring) or repressed (green) inside murine Mφs (d) [12], and up-regulated (red) or down-regulated (green) in a hollow fibre murine model (e) [47].

**Figure 7:**
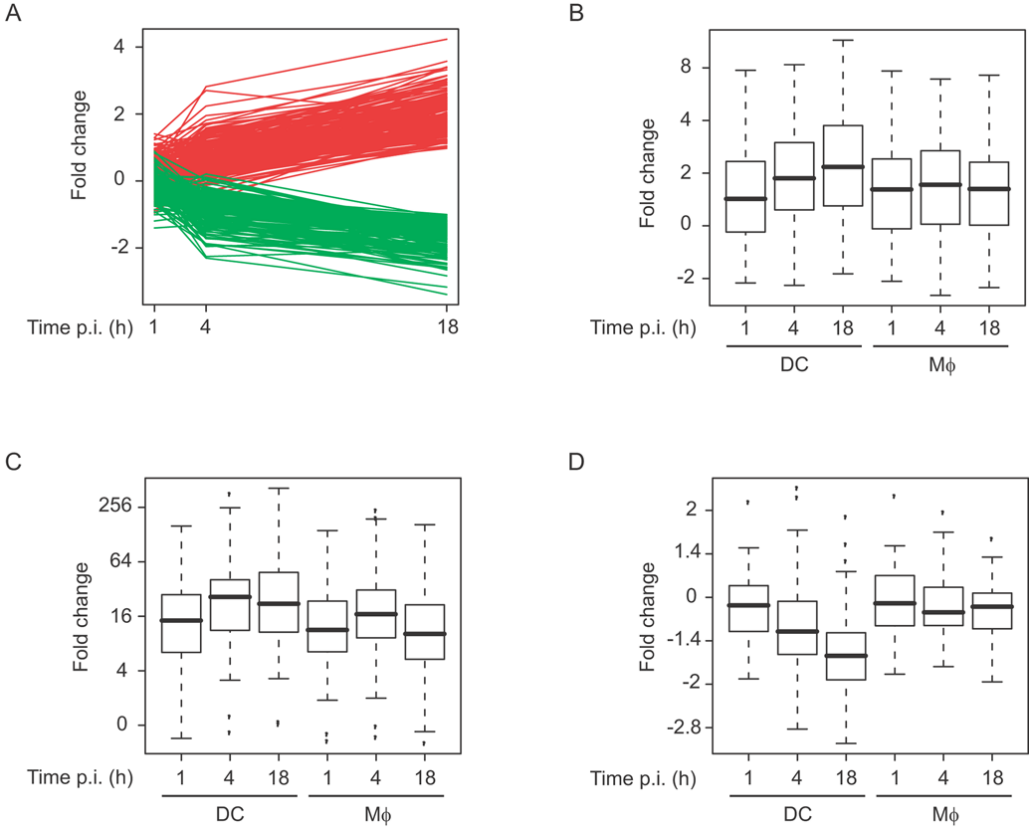
Cell-specific responses of *M. tuberculosis* to Mφs and DCs. (A) The transcriptional profiles of 191 genes (red colouring) and 153 genes (green) identified to be significantly over-expressed in DCs and Mφs respectively at 18 h post infection. Box plots showing the gene expression pattern (in fold change) of (B) *kstR* regulon [46], (C) *dosR* regulon [45], and (D) ribosomal gene family (functional category II.A.1 [63]), in DCs and Mφs at 1, 4 and 18 h timepoints relative to aerobic growth.

A core set of genes involved in the adaptation of bacilli to the intracellular environment and representative of the *in vivo* phenotype of *M. tuberculosis* was observed (Figure 6), as previously reported by others [3], [12], [13], [14], [15], [16]. The switch to a lipolytic lifestyle *in vivo* was demonstrated by the induction of genes involved in the β-oxidation of fatty acids [12] (*fadD3/9*, *fadE5/14/24/28/30/33/34*, *echA6/7/12/19/20*, *fadB2*, *fadA6*), the glyoxylate shunt [38] and gluconeogenesis (*icl*, *gltA1*, *pckA*), and cholesterol metabolism (42 genes from the previously defined gene cluster [39], hypergeometric probability 3.4×10^−15^). The changing respiratory state of the bacilli inside human phagocytes from aerobic to micro-aerobic or anaerobic was exemplified by the induction of genes involved in alternative electron transfer (*fdxA/C*, *narK2/X*) and the down-regulation of the type I NADH dehydrogenases relative to aerobic log phase growth (*nuoA-N*) [40]. A large number of these genes are co-ordinately transcribed through the *dosR/S/T* and *kstR* regulatory systems. The *dosR*/*dosS* two-component system, that allows coordinated response to several stresses including O_2_ deprivation and exposure to oxidative radicals [41], [42], [43], [44], was induced in both Mφs and DCs. Accordingly, 45 members of the *dosR* regulon [45] were up-regulated intracellularly (p = 3.3×10^−34^). Similarly over half the genes of the *kstR* regulon, predicted to be involved in lipid degradation and cholesterol utilization, were up-regulated in both cell types compared to *M. tuberculosis* aerobic growth (p = 1.0×10^−9^) [46]. The changing expression pattern of these two regulons over time in each infection model is depicted in Figure 7B–C. Genes involved in the sequestration of iron (*mbtB/D/E/F/I/J*) were also induced. Interestingly, several genes encoding enzymes or molecular partners involved in the synthesis of polyketides (*papA1*, *papA3*, *pks2-4*) were down-regulated in both DCs and Mφs. This likely reflects reorganization of the mycobacterial cell wall during infection.

Analysis of the intracellular gene expression profiles also allowed cell type-specific signatures to be identified in the mycobacterial transcriptome. Differences were in general apparent early in infection, but only became statistically significant at 18 h post-infection, when 153 and 191 genes were over-expressed in Mφs and DCs, respectively (Figures 6B & 7A). Many of the genes over-expressed in DCs were induced in both DCs and Mφs relative to aerobic growth, and were thus up-regulated in DCs to a significantly greater degree than in the *M. tuberculosis* response to Mφ infection. Genes over-expressed in DCs compared to Mφs included many members of the *dosR* and *kstR* regulons (hypergeometric p-values 1.8×10^−16^ and 2.4×10^−16^ respectively; Figure 7B–C), genes involved in amino acid biosynthesis (*argB-D*, *argF*, and *hisC/D/F*), and lipid degradation (*echA19, fabD, fadA5, fadD13/19, fadE12/23/26-28/34, mmsA, mutA-B*), as well as 24 genes belonging to the cholesterol catabolism gene cluster *Rv3492c-Rv3574* recently identified in *M. tuberculosis* [39]. The induction of genes in DCs compared to Mφs of functional significance in nitrate respiration (*narG/narK2*) and in respiration in limiting O_2_ conditions (*cydA-D*) was also demonstrated. Many genes over-expressed in DCs compared to Mφs have also been identified to be induced during dormancy *in vivo* [47] (Figure 6C), nutrient starvation [48], in limiting O_2_ conditions [49] or associated with a slowed mycobacterial growth rate [50].

Genes encoding ribosomal proteins (*rplB*, *rplF*, *rplN*, *rpsF*, *rpsN* ; Figure 7D) and DNA biosynthesis (*dnaB/N*, *fusA*) were under-expressed in DCs compared to Mφs, indicating slowed mycobacterial replication in these cells [48]. This links with the observation that the *relA* gene was over-expressed in *M. tuberculosis* extracted from DCs compared to Mφs at 18 h post-infection indicating that the stringent response may be regulated in a cell-specific manner. Many genes in the biosynthesis (*papA5*, *ppsC*, *pks1*, *pks15*, *fadD22/29*) and export (*drrB-C*, *lppX*) [51], [52] of phtiocerol dimycocerosate (PDIM), a surface molecule that is important in pathogenesis, were also more weakly expressed in the *M. tuberculosis* response to DC compared to Mφ infection.

Taken together, these results suggest that DCs restrict access of intracellular mycobacteria to important nutrients, including amino acids, and indicate that the switch of mycobacterial metabolism towards fatty acid utilization as a carbon source and micro-aerobic or anaerobic respiration is even furthered inside DCs than inside Mφs. Furthermore many of these cell type-specific changes in gene expression are also differentially regulated over time, with a large proportion of the genes that are over-expressed in DCs *vs* Mφs at 18 h also significantly induced in *M. tuberculosis* extracted from DCs at 18 h compared to 1 h post-infection (Figures 6B & 7A). We confirmed that the bacterial growth is indeed reduced in DCs (Figure 4C). Altogether, these results clearly indicate an increased mycobacterial stress response in DCs and support and extend previous findings [17] describing the limited ability of *M. tuberculosis* to multiply inside human DCs.

## Discussion

Here we have investigated host-pathogen cross-talk by profiling global gene expression over a time course in both the host cell and the microbe simultaneously, using *M. tuberculosis*-infected human Mφs and DCs as a model system. Previously, a number of important studies have been published dealing with global host cell [7], [8], [9], [10], [11] or *M. tuberculosis* [12], [13], [14], [15], [16], [40], [48], [50] gene expression profiling upon infection. This is the first time however that simultaneous host-pathogen profiles have been produced with this important pathogen. We have also used the most appropriate human cell model, and this is the first time indeed that the transcriptome of *M. tuberculosis* has been described in human DCs.

In addition to core responses, we have identified a number of cell type-specific signatures in both the mycobacterial and the host cell transcriptomes. This allowed us to extract information not only on the physiology of both host immune cells and intracellular bacilli during infection, but also on how intracellular mycobacteria perceive different environments, and how host cells respond differentially to intracellular infection.

Although the *M. tuberculosis* response to the intracellular environment measured here reflects many features of the *M. tuberculosis in vivo* phenotype [12], [15], [16], our results clearly indicate that mycobacteria respond differently to phagocytosis by the two different phagocyte types. Transcriptome analysis indicates that the bacilli perceive the DC phagosome as a more constraining environment than the Mφ phagosome, with a greater induction of stress responsive genes during DC infection. Over-expression of ribosomal genes in Mφs as compared to in DCs for instance, is an indicator of active protein synthesis and likely of bacterial division. This is in accordance with our previous results showing mycobacterial growth inside human-derived Mφs and mycobacterial stasis inside human DCs [17]. The ability of DCs to control infection by intracellular pathogens seems not to be restricted to *M. tuberculosis*, and has also been observed with other mycobacteria [53], [54] and other bacteria [55], [56], [57], [58]. This might represent a unique strategy evolved by DCs to cope with intracellular pathogens and ensure their antigen presenting functions [17]. Although the stress response observed in DCs is clear, the very nature of the stress encountered by the bacilli inside these cells is not easy to identify. Possible obvious explanations include differential reactive oxygen and nitrogen species production, phagosome acidification, and/or nutrient limitation. The results obtained from both the mycobacterial and host cell transcriptomes allow us to raise and evaluate possible hypotheses.

Genes of the *dosR* regulon were induced in both DCs and Mφs, and many of them were over-expressed in DCs. The most highly induced gene of the *dosR* regulon (*acr1*/*hspX*) has been implicated in actively slowing down bacterial growth [59], [60], so induction of this regulon would partly explain growth limitation. The *dosRST* two component system responds to O_2_ limitation and/or to NO [42], [45], [61] and our datas*et al*lows us to look for the likely stimulus by analysing the host transcriptome. Because *NOS2*/*iNOS* was not induced and NO was not detected in either cell type, it is unlikely that increased NO production explains the mycobacterial stress response observed in DCs. The possibility remains that O_2_ might be limiting in DCs. At the level of the host cell transcriptome, this might be reflected by the increased number of genes involved in respiration and energy production whose expression was modulated in DCs as compared to in Mφs (Figure 2A). An increased O_2_ consumption by DCs may account for the mycobacterial phenotype inside these cells, which should be further investigated. Furthermore the *M. tuberculosis ald* gene is over-expressed in DCs. This gene encodes a functional alanine dehydrogenase that might be involved in NAD^+^ regeneration under low O_2_ conditions [62]. Many of those genes that have been identified as being up-regulated under limiting O_2_ conditions [49] were found to be over-expressed in DCs.

Superoxide production is another possible source of bacterial stress. The observations that several genes involved in synthesis of the phagocyte oxidase, such as *gp91^phox^*, were down-regulated in DCs upon infection, and the global superoxide production was less in DCs than in Mφs, argue against this, although differential assembly of the enzyme complex at the phagosome membrane may lead to an increased O_2_^−^ production locally in the mycobacterial vacuole. Interestingly, and opposite to what is observed in Mφs, infected DCs seem to preferentially synthesise Rac1 rather than Rac2. This differential synthesis of Rac isoforms may have important consequences on the level of superoxide production [25]. The exact topology and activity of the phagocyte oxidase at the phagosome membrane in DCs and Mφs should be further studied and compared. In addition, 18 oxidoreductases (including *adhD* and *hmp*) and three cytochrome P450s (*cyp125/132/144*) were over-expressed in *M. tuberculosis* extracted from DCs at 18 h compared to Mφs; furthermore the functional category of I.B.7. (miscellaneous oxidoreductases and oxygenases [63] was identified to be significantly enriched (p = 1.4×10^−3^). Seven of these probable oxidoreductases were predicted to be part of the *kstR* regulon [46], other members of which were also identified to be over-expressed in the *M. tuberculosis* response to DCs compared to Mφs infection. The cell-specific induction of these genes that may be involved in the mycobacterial response to oxidative stress may reflect the increasingly hostile environment encountered by bacilli inside DCs at 18 h post infection, compared to Mφs.

We believe that a third possibility to explain the constrained phenotype of mycobacteria in DCs-altered phagosome acidification - is unlikely because the phagosomal lumen is kept slightly alkaline in DCs, which allows moderate antigen degradation and proper antigen presentation [27]. We have previously reported that the mycobacterial phagosome was not more acidic in DCs than in Mφs [17].

A fourth possibility which would deserve further exploration in future studies is differential nutrient starvation. Several mycobacterial genes involved in nutrient uptake, storage or synthesis were over-expressed in DCs. This is the case, for instance, of the glutamine uptake gene *glnH*, which might indicate limited mycobacterial access to iron and glutamine in DCs. Previous work has shown that some genes involved in glutamine synthesis are essential *in vitro* and *in vivo* [64], [65], [66]. A large number of genes (45) over-expressed in DCs have previously been reported to be induced in nutrient limitation conditions (*e.g. pdhA/B*, *cysD*, *ald*, *lat*, and *rocA/D1*) [48], in addition *relA*, which encodes the putative regulator of the stringent response that is induced under starvation, was also over-expressed during DC compared to Mφ infection.

In relation to nutrition, our results strongly suggest differential utilization of cholesterol by mycobacteria in DCs and Mφs. Indeed, 24 genes belonging to the recently identified cholesterol catabolism gene cluster [39] were found to be significantly over-expressed in DCs ; 23 of these have been identified as part of the *kstR* regulon [46]. The functional characterisation of this large regulon and the role of cholesterol utilization by intracellular mycobacteria is still elusive, and requires further exploration. It is likely that KstR, a transcriptional repressor, is bound by lipid molecules (perhaps cholesterol or a cholesterol derivative), so inducing the regulon that degrades these lipids for energy and as a carbon source. Thus the fact that the regulon is activated more rapidly in DCs indicates the presence of these nutrients, and perhaps the lack of an alternative carbon source.

In conclusion, our study provides a solid framework to be used to further understand host cell-mycobacteria interactions, and paves the way for a number of novel investigation questions. This unique dataset will be increasingly useful as more functional information and regulatory networks are defined, and can be interrogated as needed. Although known before that mycobacterial growth is limited in DCs in comparison to Mφs, the mechanisms are not understood. Our data provide a detailed picture that will allow mechanisms to be proposed and tested. These results underline the extraordinary plasticity of the mechanisms involved in the host cell response to infection and of microbial adaptation to different intracellular environments. It is a subtle and dynamic picture that emerges. Such a fine tuning in molecular responses likely results from long periods of co-evolution ; further understanding these mechanisms should ultimately lead to novel and adapted intervention strategies to combat TB and other deadly infectious diseases.

## Materials and Methods

### Bacteria, cells and infection

Human monocytes were purified from cytapheresis rings and differentiated into Mφs or DCs according to a previously described procedure [17]. *M. tuberculosis* H37Rv was grown from a frozen stock to mid-log phase in 7H9 medium (BD) supplemented with albumin-dextrose-catalase (ADC, Difco). The intact virulence of bacteria in the frozen stock was checked by infecting C57BL/6 mice intranasally with 10^3^ bacilli. After 21 and 42 days, the bacterial load in the lungs was of about 10^7^ bacteria. Cells were infected as previously described [17], at multiplicities of infection (MOI) of 5 and 2 for the 1 h and 4 h/18 h time-points, respectively, for mycobacterial RNA extraction ; and at a MOI of 1 for cellular RNA extraction. After 1, 4, 18 h and 48 h of infection, cells were recovered by centrifugation and processed for cellular or bacterial RNA extraction.

### Cellular RNA extraction, preparation, and hybridisation to the arrays

Total RNA was extracted from all 9 individual donors (corresponding to 72 samples in total) using TRIZOL® reagent (Life Technologies Inc., Carlsbad, CA) and further purified with RNeasy columns (Qiagen, Valencia, CA), as described by the manufacturers. All sample quality was controlled strictly to verify the RNA integrity before use in microarray experiments. RNA quantity was evaluated spectrophotometrically, and the quality was assessed with the Agilent 2100 bioanalyzer (Agilent Technologies Inc, Palo Alto, CA). Only samples with good RNA yield and no RNA degradation (28S∶18S>1.7 and RNA integrity number>8.5) were retained for further experiments. Labelling of samples and hybridisation to the Human U133A oligonucleotide microarray chips (Affymetrix, Santa Clara, CA) containing 22,283 probe sets were performed according to the manufacturer's protocols. Briefly, for the microarray experiment, 10 µg of total RNA was used for cRNA synthesis. cDNA was synthesized *in vitro* with the BioArray HighYield RNA Transcript Labeling kit (Enzo Life Sciences, Farmingdale, NY) with a T7-(dT)24 primer for this reaction. Biotinylated cRNA was then generated from the cDNA reaction using the BioArray High Yield RNA Transcript Kit. The cRNA was then fragmented in fragmentation buffer (5× fragmentation buffer: 200 mM Tris-acetate, pH 8.1, 500 mM KOAc, 150 mM MgOAc) at 94°C for 35 min before the chip hybridisation. Fifteen micrograms of fragmented cRNA was then added to a hybridisation cocktail (0.05 µg/µL fragmented cRNA, 50 pM control oligonucleotide B2, BioB, BioC, BioD, and cre hybridisation controls, 0.1 mg/mL herring sperm DNA, 0.5 mg/mL acetylated BSA, 100 mM MES, 1 M NaCl, 20 mM EDTA, 0.01% Tween 20) and cRNA was hybridised to chips. The arrays were washed and stained with R-phycoerythrin streptavidin in the GeneChip Fluidics Station 400. The arrays were then scanned with the GeneArray Scanner. Affymetrix GeneChip Microarray Suite 5.0 software was used for washing, scanning, and basic analysis. Following scanning, array images were assessed by eye to confirm scanner alignment and the absence of significant bubbles or scratches. 3′/5′ ratios for glyceraldehyde-3′-phosphate dehydrogenase (GAPDH) and β-actin were confirmed to be within acceptable limits range from QC report, and BioB spike controls were found to be present on 100%, with BioC, BioD and CreX also present in increasing intensity. When scaled to a target intensity of 150 scaling factors for all arrays were within acceptable limits as were background, Q values and mean intensities.

### Bacterial RNA extraction, preparation, and hybridisation to the arrays

Mycobacterial RNA was extracted from infected Mφs or DCs by a differential lysis procedure using the GTC/Trizol method developed by Mangan *et al.* [12], [37]. RNA was DNase-treated and purified using RNeasy columns (Qiagen), and quantified using the NanoDrop ND-1000 Spectrophotometer (NanoDrop Technologies) and Agilent 2100 Bioanalyser (Agilent Technologies). 250 ng total *M. tuberculosis* RNA was amplified using an Eberwine T7-oligo-dT-based system after an initial polyadenylation step (MessageAmp II Bacteria, Ambion). Single rounds of amplification were performed, with an IVT reaction of 16 hours at 37°C. This amplification method has been previously demonstrated to be reproducible and capable of identifying representative changes in gene expression (Waddell *et al.* Submitted manuscript). Microarray hybridisations were conducted as previously described [67] with 5 µg Cy5-labelled cDNA derived from amplified *M. tuberculosis* RNA against 2 µg Cy3-labelled *M. tuberculosis* H37Rv genomic DNA. Mycobacterial RNA was extracted from Mφs and DCs from three healthy donors at 1, 4 and 18 h post infection, and from two biological replicates of log phase *in vitro* growth, and hybridised in duplicate to a *M. tuberculosis* whole genome microarray (ArrayExpress accession number A-BUGS-23, http://bugs.sgul.ac.uk/A-BUGS-23).

### Cellular microarray data analysis

The following analysis steps were performed using a modified version of the AMDA library [68]. Four quality checks were performed to verify the quality of sample preparation and hybridisation. These are based on the frequency of probe sets with Detection call ‘Absent’ or ‘Present’ and their associated averaged values in each sample, as well as on the ratios between the expression values for 3′ and 5′ end of *Gapdh* and *Actin* transcripts. All the resulting values were in agreement with the highest Affymetrix recommended quality standards.

Probe-level background corrected expression intensities were generated starting from the image files following the Affymetrix recommendations as implemented in the GCOS software. Probeset-level were generated using the GCRMA method [69] and normalized using quantile normalization [70] at the probe level.

To filter out noisy data before the selection of differentially expressed genes, a filter was applied based on Detection calls. As a first step, probe sets called ‘Absent’ over all conditions and replicates were removed (5,792). As a second step, the 95^th^ percentile of all the signals of the entire dataset that were flagged with an absent call was determined and used as a threshold to remove all the remaining probe sets whose expression values were always below this value in each sample (5,229). Finally 11,262 probe sets remained for the next analysis steps.

Hierarchical clustering based on *complete linkage* method and Pearson correlation as a similarity measure was applied to evaluate the effect of the different sources of variability (donors, time-points and host specific responses). The resulting dendrogram can be interpreted similarly to a phylogenetic tree and the vertical scale indicates 1 - Pearson correlation coefficients as a measure of similarity.

The Limma Bioconductor library developed by Gordon Smyth *et al.* [71] was used for the detection of differentially expressed genes. This method is based on the fitting of a linear model to estimate the variability in the data. In case of one-channel microarray data this approach is the same as analysis of variance except that a model is fitted for every gene. A statistics of differential expression for the analysis of paired data (pairing based on donors) is used and an empirical Bayes method is applied to moderate the standard errors. Differentially expressed genes have been selected based on a threshold p-value of 10^−4^. P-values have been corrected for multiple testing using Benjamini & Hochberg's method to control the false discovery rate.

A functional annotation of differentially expressed genes was performed on the basis of the annotation provided by the HGU133a Bioconductor library (version 1.14.0). In particular we focused on the annotation available from Gene Ontology (GO, www.geneontology.org, [71]) and KEGG (www.genome.jp/kegg, [71]).

Only the functional categories with at least 3 differentially expressed genes have been considered. The most representative functional annotations for each experimental condition are identified using the hypergeometric distribution to determine the probability of random occurrence of functional terms (functional enrichment). Based on this probability ranking only the top 50 statistically most significant annotation terms are reported. To perform a statistical test not biased by the redundancy of the probe sets (more probe sets for the same gene), the computation of p-value of functional enrichment was based on the Entrez Gene assignment. Functional annotations have been clustered based on the Log_10_ of enrichment p-values of different annotation terms across the different conditions. This plot can be useful to compare the functional characterization of differentially expressed genes in the different conditions. In particular annotation terms specific for a subset of conditions could be identified, as well as annotation terms that are equally relevant for all the conditions. Note that the colours of such a graph (Figure 2) reflect only the enrichment p-values (highly significant is red), they are not representative of the direction of the modulation (up/down-modulated).

### Bacterial microarray data analysis

The hybridised slides were scanned sequentially at 532 nm and 635 nm corresponding to Cy3 and Cy5 excitation maxima using the Affymetrix 428™ Array Scanner (MWG). Comparative spot intensities from the images were calculated using Imagene 5.5 (BioDiscovery) and data from multiple scans combined using MAVI 2.6.0 software (MWG Biotech AG). Data analysis was performed using functions from the Limma (linear models for microarray data analysis) software package [72]. The array data were filtered to include only cDNA elements flagged to be present on 80% of the arrays, and normalised to the 50th percentile of all remaining genes. Differentially expressed genes were identified using a “Two-Groups: Common Reference” experimental design. Duplicate spots within arrays and across technical replicate hybridisations (*i.e.* all replicate values for the same RNA sample) were averaged before performing the linear model fit. Genes with a moderated t-test p value (with Benjamini and Hochberg multiple testing correction) of<0.05 were considered to be significantly differentially expressed. These genes were hierarchically clustered using Cluster and the results displayed using Treeview software [73]. The hypergeometric distribution was used to determine if functional categories of genes were significantly enriched in the intracellular profiles [74].

### Quantification of superoxide production

Superoxide production by PMA-treated or *M. tuberculosis*-infected cells was quantified using the Lumimax® Superoxide Anion Detection kit (Stratagene, La Jolla, CA) following the manufacturer's instructions.

### Analysis of cellular gene expression with Western blotting

Cellular proteins were quantified using the Micro BCA™ kit (Pierce, Rockford, IL). For each extract analyzed, 10 µg of proteins were loaded per well, and proteins were detected using anti-Rac1 (BD Biosciences, San Jose, CA) and -Rab9A (Abcam Inc., Cambridge, MA) monoclonal antibodies.

### Access to microarray data

Gene regulation data for all analyzed eukaryotic genes are available in the publicly available GENOPOLIS database (https://gc-lab32.btbs.unimib.it/genopolisDB/html/users.php; login: Olivier.Neyrolles ; password: genopolis). Fully annotated raw and filtered *M. tuberculosis* microarray data has been deposited in BµG@Sbase (accession number: E-BUGS-58; http://bugs.sgul.ac.uk/E-BUGS-58) and also ArrayExpress (accession number: E-BUGS-58). Both databases are MIAME compliant. Preliminary access to the *M. tuberculosis* dataset is available at http://bugs.sgul.ac.uk/bugsbase using the username: journalaccount3, password: hg67Ky42B. To view microarray experiment details select E-BUGS-58 from the drop-down menu, then Find. Then click on the experiment summary tree to access protocols and raw/filtered expression data.

## Supporting Information

Figure S1 Mean expression levels of selected genes (in arbitrary units, a.u.) in *M. tuberculosis*-infected human macrophages (open circles) and dendritic cells (filled circles).
(0.48 MB EPS)
